# Impact of perioperative use of GnRH agonist or dienogest on ovarian reserve after cystectomy for endometriomas: a randomized controlled trial

**DOI:** 10.1186/s12958-021-00866-2

**Published:** 2021-12-06

**Authors:** Ayako Muraoka, Satoko Osuka, Atsushi Yabuki, Bayasula, Masato Yoshihara, Hideaki Tanaka, Reina Sonehara, Natsuki Miyake, Mayuko Murakami, Sayako Yoshita, Natsuki Nakanishi, Tomoko Nakamura, Maki Goto, Akira Iwase, Hiroaki Kajiyama

**Affiliations:** 1 Department of Obstetrics and Gynecology, Nagoya University Graduate School of Medicine, 65 Tsurumai-cho, Showa-ku, 466-8550 Nagoya, Japan; 2 Bell Research Center for Reproductive Health and Cancer, Nagoya University Graduate School of Medicine, 65 Tsurumai-cho, Showa-ku, 466-8550 Nagoya, Japan; 3 Department of Obstetrics and Gynecology, Gunma University Graduate School of Medicine, 3-39-22 Showa-machi, 371-8511 Maebashi, Japan

**Keywords:** Anti-Müllerian hormone, Cystectomy, Endometriomas, Ovarian reserve

## Abstract

**Background:**

Ovarian endometrioma is a common gynecological disease that is often treated with surgery or hormonal treatment. Ovarian cystectomy, a surgical procedure for ovarian endometrioma, can result in impaired ovarian reserve.

**Methods:**

We conducted a randomized controlled trial to evaluate the efficacy of hormonal treatment [gonadotropin-releasing hormone agonist (GnRHa) or dienogest (DNG)] for preserving ovarian reserve after cystectomy for ovarian endometrioma. The primary endpoint was the level of serum Anti-Müllerian hormone (AMH) as a marker of ovarian reserve.

**Results:**

Before and after laparoscopic surgery, 22 patients in the GnRHa group and 27 patients in the DNG group were administered hormonal treatment for a total of 4 months. After 1-year follow-up, >60% of the patients in the DNG group retained over 70% of their pretreatment AMH levels, whereas no patient in the GnRHa group retained their AMH levels after cystectomy (*P* < 0.01). Interleukin-6 (IL-6) is a key cytokine involved in inflammation. Compared with the GnRHa group, patients in the DNG group had lower IL-6 levels at the end of treatment.

**Conclusions:**

Our data revealed that DNG is more effective than GnRHa in preserving ovarian reserve after cystectomy of ovarian endometrioma. This is achieved through the reduction of the inflammatory response during the perioperative period and other endometriosis-related inflammatory reactions.

**Trial registration:**

The registration number of this trial is UMIN-CTR, UMIN000018569, registered 6 August 2015, https://upload.umin.ac.jp/cgi-open-bin/ctr_e/ctr_view.cgi?recptno=R000021492, and Japan Registry of Clinical Trials, jRCTs041180140, registered 29 March 2019, https://jrct.niph.go.jp/en-latest-detail/jRCTs041180140. This randomized controlled trial was conducted in accordance with the CONSORT guidelines.

**Supplementary Information:**

The online version contains supplementary material available at 10.1186/s12958-021-00866-2.

## Background

Endometriosis is the development of endometrial tissue outside the uterus, which causes pelvic pain and infertility [1]. Although endometriosis is a benign disease, severe clinical symptoms of endometriosis compromise the quality of life of women of reproductive age. Hormonal treatment is an effective therapeutic strategy for endometriosis; however, surgical treatment, usually through ovarian cystectomy, is required to manage hormonal treatment-resistant symptom [1]. Although cystectomy is one of the most commonly used approaches for the treatment of ovarian endometrioma, endoscopic management is still controversial especially for women who plan to become pregnant. Several studies have suggested that ovarian reserve is compromised after laparoscopic cystectomy for ovarian endometrioma management [2, 3].

Ovarian reserve is defined as the functional potential of the ovary which reflects the number and quality of the remaining follicles [4]. Anti-Müllerian hormone (AMH) is a reliable serum marker of ovarian reserve and is produced by the granulosa cells of primary to small antral follicles to prevent depletion of the primordial follicle pool [5]. Serum AMH levels are preferred in determining the indication and selection of operative methods for benign gynecologic conditions, especially for the management of endometriomas [6–11].

Preoperative gonadotropin-releasing hormone agonist (GnRHa) and dienogest (DNG) treatments have been generally accepted as a method to improve the painful symptoms before surgery and to simplify laparoscopic surgery by reducing the size of the endometriomas and suppressing inflammation [12, 13]. Several previous studies have demonstrated that preoperative hormonal treatment improves the symptoms before surgery; however, the surgical outcomes are not influenced by the administration of the preoperative hormonal treatment [14–16]. There have been no studies evaluating the impact of hormonal treatment on ovarian reserve after cystectomy for a long follow-up period.

Therefore, the present study was conducted to determine whether hormonal therapy affects the ovarian reserve after cystectomy for the management of ovarian endometrioma. The study is specifically focused on discerning which hormonal treatment is more effective in ovarian reserve preservation through the measurement of the serum AMH levels.

### Methods

### Patients

This study was approved by the Institutional Review Board of the Nagoya University Graduate School of Medicine (no. 2015-0288), registered UMIN-CTR (UMIN000018569, date of registration 08/06/2015, https://upload.umin.ac.jp/cgi-open-bin/ctr_e/ctr_view.cgi?recptno=R000021492) and Japan Registry of Clinical Trials (jRCTs041180140, date of registration 03/29/2019, https://jrct.niph.go.jp/en-latest-detail/jRCTs041180140), and was conducted in accordance with the principles of the Declaration of Helsinki. Informed consent was obtained from all patients. All patients had regular menstrual periods and did not receive any hormonal treatment for at least three months before admittance to the study. This study was conducted from June 2016 to May 2020 in the Department of Obstetrics and Gynecology of Nagoya University Hospital in Nagoya, Japan. Prior to enrollment, all patients were previously diagnosed with endometriomas by transvaginal ultrasound examinations and magnetic resonance imaging (MRI). The inclusion criteria included: [1] women aged 20-42 years with regular menstrual cycles (25-35 days), and [2] patients who were diagnosed with ovarian endometrioma larger than 4 cm in diameter by transvaginal ultrasound examinations and MRI. The exclusion criteria included [1] a previous history of ovarian or adnexal surgery, [2] suspicious findings of malignant ovarian diseases, or [3] evidence of any other endocrine disorders, including thyroid dysfunction, hyperprolactinemia, Cushing syndrome, and polycystic ovarian syndrome (PCOS). This randomized controlled trial was conducted in accordance with the CONSORT guidelines.

### Randomization and hormonal treatment

**Fig. 1:**
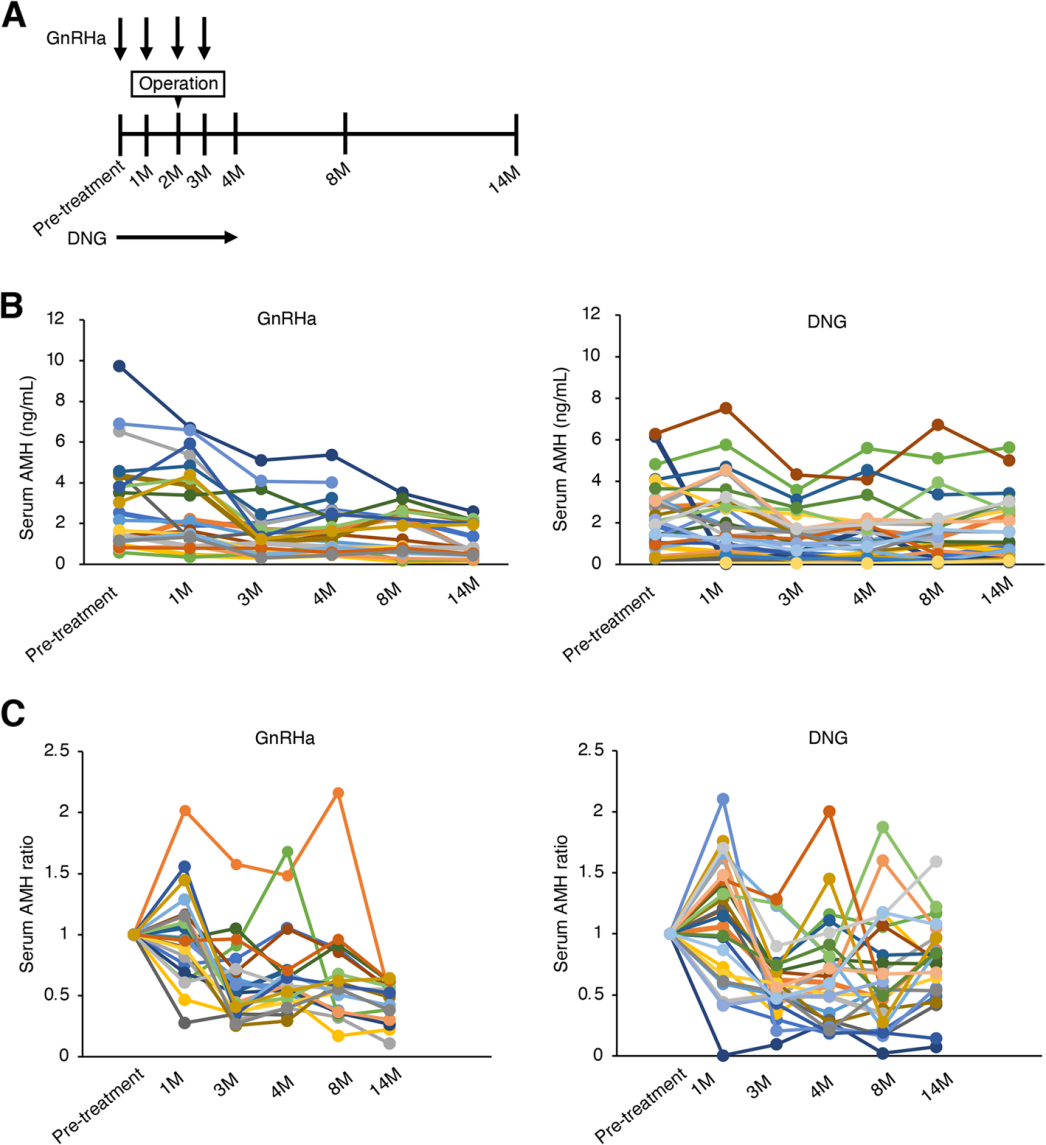
Changes in AMH levels. (**A**) Schematic presentation of treatment protocol and timing of blood tests. (**B**, **C**) The serial changes in the serum AMH levels (**B**) and AMH ratios (**C**) in each case in the GnRHa group and DNG group

After enrollment to the study, the patients were arbitrarily categorized into the GnRHa or DNG groups according to a randomization table generated using a software. Patients in the DNG group received dienogest (Dinagest®, 1 mg; Mochida Pharmaceutical Co., Ltd., Tokyo, Japan) at 2 mg/day for 2 months preoperatively and 2 months postoperatively. Patients in the GnRHa group received buserelin acetate (Suprecur MP®, 1.8 mg; Mochida Pharmaceutical Co., Ltd., Tokyo, Japan) at 1.8 mg/month total administrations pre- and post-operatively. A schematic representation of the treatment protocol and timing of blood tests is shown in Fig. 1 A.

### Surgery

All patients underwent laparoscopic surgery under general anesthesia [6]. The wall of the cysts was stripped from the surrounding normal ovarian tissue using two atraumatic grasping forceps by traction and countertraction after identification of the cleavage plane. When necessary, hemostasis was achieved with bipolar forceps which were minimally utilized to avoid damaging normal tissues. The hemostasis time using bipolar forceps was measured by retrospectively analyzing the surgical videos. Suturing was performed for all patients for closure of the ovarian parenchyma but not for hemostasis. Endometriosis was classified according to the revised American Society for Reproductive Medicine (rASRM) classification during surgery. All laparoscopic procedures were performed by the same surgical team.

### AMH Measurements

Blood samples were obtained from the patients 1 and 2 months before surgery and at 1, 2, 6, and 12 months after surgery. Serum was separated from the whole blood and stored at -80 °C until the assay analysis. The serum AMH concentrations were measured using an enzyme immunoassay kit according to the manufacturer’s instructions (Elecsys AMH Plus; Roche).

### Cytokine analysis

The patients’ sera were used for cytokine array (Human Cytokine Arrays C3; RayBiotech) according to the manufacturer’s instructions. Dot blots analysis was facilitated using a circular region to quantify each pair of dots and the corresponding mean was obtained. The LAS4000 CCD-Imaging System (Fujifilm Co. Ltd., Tokyo, Japan) was used to detect proteins.

### Chemiluminescent enzyme immunoassay (CLEIA)

Serum concentrations of IL-6 were measured using the CLEIA method (FUJIREBIO, Tokyo, Japan) at an outsourced laboratory (SRL, Tokyo, Japan).

### Histological analysis

For ovarian tissue removal assessment, we confirmed the resection of either primordial, primary, secondary, or Graafian follicles in pathological slides of specimens from each patient using an optical microscope (BX60; Olympus Corporation).

### Statistical analysis

According to the statistical analysis of our pilot study, it was determined that 30 patients in each group would provide the trial with 80% power at a two-sided significance level of 0.05. This showed a significant difference in AMH levels between the GnRHa and DNG groups, with a corresponding 40% reduction of AMH in the GnRHa group and 20% reduction in the DNG group after cystectomy. Because the reduction rate of the primary endpoint (AMH level after 12 months from cystectomy) was lower than projected, the analysis was performed when 57 patients were enrolled. Microsoft Excel and R were used to generate graphs and perform statistical analyses. Data are presented as the mean ± standard deviation (SD). The *P* -values were calculated using a two-sided Student’s t-test or the Mann–Whitney U test for continuous variables, whereas categorical variables were compared using the Chi-square test or Fisher’s exact test. Statistical significance was set at *P* <0.05. The *P*-values of statistical significance were indicated as n.s., *, *P*<0.05; **, *P*<0.01.

## Results

**Fig. 2:**
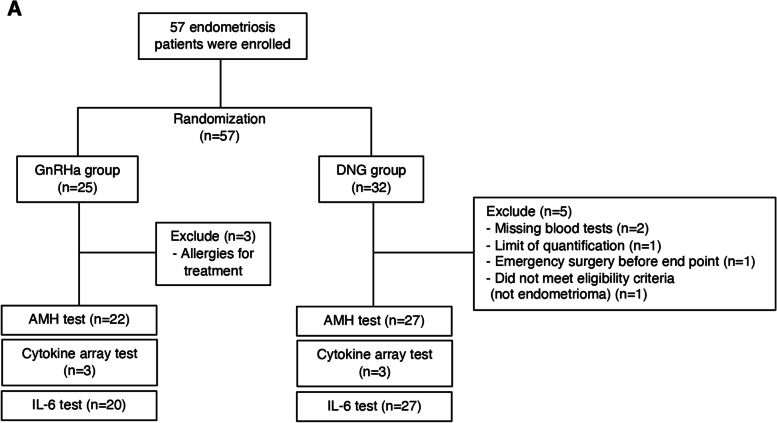
RCT chart. (**A**) Flowchart of the process for the RCT of hormonal treatment. A total of 57 patients were enrolled in this study and were correspondingly divided into two groups

**Fig. 3:**
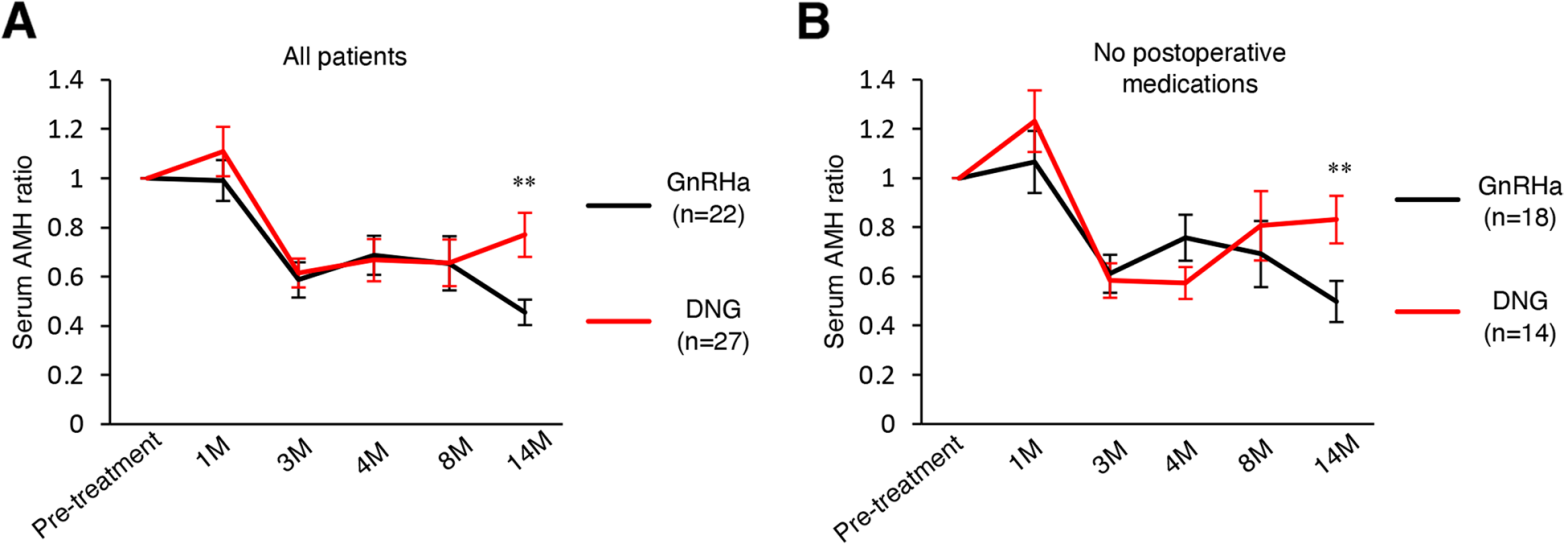
Comparison of serial changes in AMH ratios between the GnRHa and DNG groups. (**A**,**B**) The serial changes in the serum AMH ratios in all patients (**A**) and in patients without hormonal treatment for 4 months (**B**). Error bars indicate SD, ** *P*<0.01. Data were analyzed by two-tailed Student’s *t*-test

A total of 57 patients were recruited, of whom 25 were treated with GnRHa and 32 were treated with DNG following randomization (Fig. 2 A). Three patients were excluded from the GnRHa group because they were allergic to the GnRHa treatment. Blood tests were missed for 2 patients in the DNG group at the time of enrollment, one patients’ value of AMH was lower the limit of quantification, one patient underwent emergency surgery (micro rupture of endometrioma) after 1 month of enrollment, and one patient did not meet the eligibility criteria for pathological diagnosis (postoperative pathological specimen did not match endometriosis), therefore, these 5 patients from the DNG group were excluded. The baseline characteristics were proportionate between the trial groups (Table 1). At baseline, there were no statistically significant differences in age, size of endometrioma, surgical parameters, and pregnancy rate after surgery (*P*>0.05). In terms of ovarian damage during surgery, there was no significant difference in the total time of hemostasis using bipolar forceps and the percentage of patients who had complications with resection of follicles by cystectomy between the two groups (*P*>0.05). Moreover, suturing was performed for all patients for closure of the ovarian parenchyma but not for hemostasis. However, serum follicle stimulating hormone (FSH) levels showed statistically significant differences 2 and 12 months after surgery between the two groups (*P*<0.05). Serum AMH levels did not show statistically significant differences between the two groups because of the large difference in values between individuals; therefore, we calculated and compared the increase or decrease in the AMH levels of each individual as a ratio based on the AMH levels at the time of enrollment. Sequential changes in serum AMH levels and ratios after enrollment were recorded (Fig. 1B C). The serum AMH ratios at 1 year after surgery were significantly higher in the DNG group than those in the GnRHa group (Fig. 3 A). There were 4 patients in the GnRHa group and 13 patients in the DNG group, who wanted to continue DNG treatment after the trial period concluded. Even when analyzed in a subgroup of patients without post-trial medication to account for drug effects, AMH ratios at 1 year after surgery were higher in the DNG group than in the GnRHa group (Fig. 3B).

**Table 1:** Patient characteristics

	Overall (n=57)	GnRH (n=22)	DNG (n=27)	*P* value
Age [years]	33.0 ± 5.6	33.0 ± 5.7	33.0 ± 5.5	0.83 *a*
Cyst size [cm]	7 [5-8]	5.5 [4.6-8]	7 [5.2-8]	0.26 *b*
Surgery				
Unilateral / Bilateral [n (%)]	34 (59) / 23 (41)	12 (54) / 10 (45)	17 (62) / 10 (38)	0.57 *c*
rASRM score	57 [36-78]	48 [37-64]	63 [37-85]	0.35 *b*
Operation time [min]	130 ± 39	123 ± 38	135 ± 39	0.28 *a*
Blood loss [ml]	51 [4.5-204]	50 [4.5-138]	95 [8.5-216]	0.31 *b*
Total time of use of hemostasis [s]	16 ± 10	12 ± 8.9	18 ± 10	0.32 *a*
Patient number of resection of follicles in specimens [n (%)]	21 (40)	8 (36)	13 (43)	0.77 *d*
Serum FSH [mlU/mL]				
pre-treatment	6.0 [4.8-7.2]	5.8 [4.9-8.4]	6.2 [4.5-6.9]	0.81 *b*
postoperative 2 month (4 M)	5.8 [3.9-7.1]	4.8 [3.1-5.7]	6.6 [4.5-7.2]	0.018 *b*
postoperative 6 month (8 M)	5.7 [4.8-9.0]	4.6 [3.8-10.2]	5.5 [4.9-7.4]	0.64 *b*
postoperative 1 year (14 M)	6.5 [4.2-7.9]	8.5 [6.7-11.4]	5.9 [3.8-9.1]	0.014 *b*
Serum AMH [ng/mL]				
pre-treatment	2.3 [1.1-3.8]	2.3 [1.1-4.0]	2.2 [1.1-3.2]	0.54 *b*
postoperative 2 month (4 M)	1.5 [0.63-2.0]	1.5 [0.84-2.1]	1.3 [0.54-2.0]	0.43 *b*
postoperative 6 month (8 M)	1.1 [0.56-2.0]	1.0 [0.68-2.1]	1.2 [0.42-2.0]	0.69 *b*
postoperative 1 year (14 M)	1.0 [0.50-2.3]	0.79 [0.48-2.2]	1.3 [0.64-2.7]	0.10 *b*
	Overall (n=32)	GnRH (n=15)	DNG (n=17)	*P* value
Pregnancy [n (%)]	10 (31)	6 (40)	4 (23)	0.45 *d*

Note: The values are presented as the mean ± standard division (SD) or median [25th, 75th percentile]

a. Student’s t-test

b. Mann-Whitney U test

c. Chi-square test

d. Fisher’s exact test

**Fig. 4:**
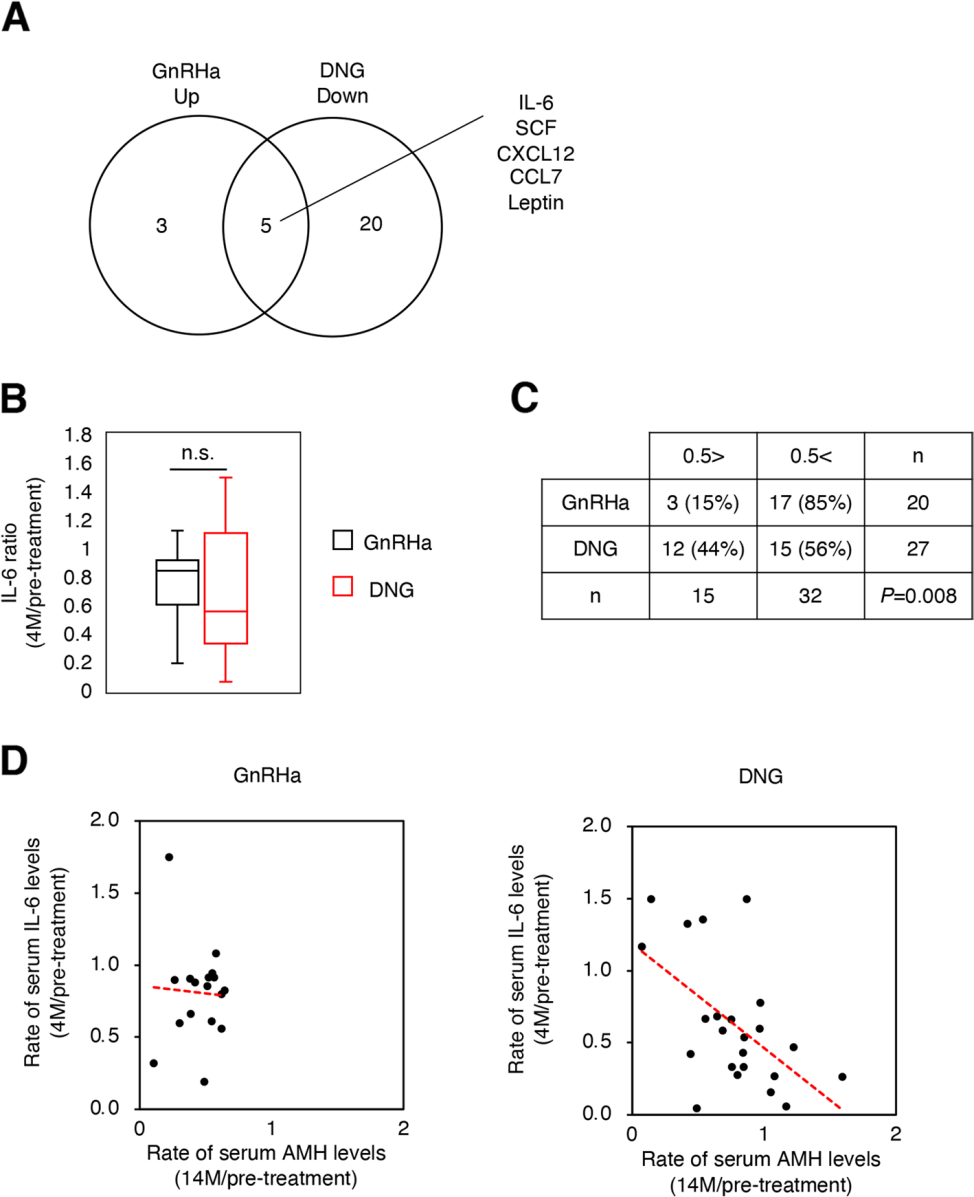
Identification of relevant factor by cytokine array. (**A**) Venn diagram of cytokines which were up-regulated in more than two out of three patients in the GnRHa group and down-regulated in more than two out of three patients in the DNG group in comparison with the time point of entry and the time point of 4 months of treatment. The roster of cytokines that are common in both groups is shown on the right side. (**B**) The ratio of serum IL-6 levels is defined as [4 M post-treatment IL-6 level / pre-treatment IL-6 level]. In the box plot, center lines show medians; box limits indicate the 25th and 75th percentiles; whiskers extend 1.5 times the interquartile range from the 25th and 75th percentiles. n.s. stands for not significant. Data were analyzed by two-tailed Student’s *t*-test. (**C**) The number of patients whose ratio of serum IL-6 levels was under 0.5 or over 0.5 in each group. The *P-*value was calculated using the Chi-square test. (**D**) Scatter plot showing the correlation between AMH and IL-6 rate in each group. (GnRHa; *r*=-0.05, *P*<0.01, DNG; *r*=-0.77, *P*=0.23) The *r* is the Pearson correlation coefficient

Endometriosis is associated with an inflammatory peritoneal environment [17]. The cytokines and growth factors were examined in response to hormonal treatment for endometriosis using a cytokine and growth factor antibody array. The serum levels of cytokines and growth factors were compared at the time of admittance and two months after surgery, as the study intended to investigate the effects of hormonal treatment during medication intake. Among the 42 cytokines and growth factors, there are five were common cytokines upregulated in the GnRHa group and downregulated in the DNG group (Fig. 4 A). IL-6 is a significantly correlated inflammatory cytokine with hormonal treatment changes. Further examination of the serum IL-6 levels was conducted using CLEIA and it was observed that the DNG group showed lower levels of IL-6 after 4 months of hormonal treatment (Fig. 4B). The ratio was calculated by serum IL-6 levels after 4 months of hormonal treatment divided by the serum IL-6 levels at the time of enrollment. When the cutoff value was set at 0.5, the number of patients in the DNG group whose ratio of serum IL-6 levels was less than 0.5 was significantly higher than that of the GnRHa group (*P*<0.01, Fig. 4 C). Furthermore, the changing status of AMH and IL-6 levels demonstrated a direct correlation with the DNG group but not statistically significant (Fig. 4D).

## Discussion

In this study, a novel insight was reported regarding the functional mechanism of perioperative hormonal treatment related to ovarian reserve preservation after endometrioma cystectomy. In the comparison of the AMH levels at the time of entry and 1 year after surgery, the serum AMH levels at 1 year were higher in the DNG group than those in the GnRHa group. Comprehensively, 17 (100%) of the 17 patients in the GnRHa group exhibited decreased AMH levels, whereas 5 (22%) of 23 patients in the DNG group exhibited increased AMH levels compared to pre-treatment levels. Moreover, none of those in the GnRHa group was able to maintain 70% of the level of AMH 1 year after surgery, while 14 (60%) of 23 patients in the DNG group were able to maintain over 70% of the level of AMH 1 year after surgery compared to pre-treatment levels.

First, we believe that preoperative hormonal therapy will improve the surgical procedure. A previous report suggested that preoperative hormonal therapy (GnRHa and danazole) affects the complexity of stripping the capsule and the associated loss of follicle [15]. However, that study’s criteria for preoperative treatment were biased in the case selection with an unstandardized preoperative hormonal therapy period. Ozaki et al. reported that symptoms related to endometriosis were completely decreased after administration of DNG compared to those in GnRHa; however, surgical outcomes such as surgical duration and blood loss were not significantly different between the two groups [18]. Based on the findings from this previous study and the concurrent findings from the present study, it can be concluded that the use of DNG as a preoperative hormonal treatment has no significant benefit to improve surgical accessibility compared to that in GnRHa. Additionally, the benefit of DNG treatment on ovarian reserve preservation is not due to reduced tissue damage during cystectomy, especially loss of follicles by the striping technique.

Endometriosis is known to be related to inflammatory responses [17]. Previous reports have shown that several inflammatory cytokines, such as IL-6, interleukin-8 (IL-8), and tumor necrosis factor-α (TNFα), were significantly elevated in the peritoneal fluid of patients with endometriosis [19]. Patients with endometriosis are constantly exposed to inflammation and even postsurgical inflammatory response could be another reason for the damage to the ovarian reserve. In this study, the relationship was analyzed between the serum AMH maintenance rate and the decreased ratio of IL-6 in each hormonal group. We focused on IL-6 because it is an inflammatory cytokine with lower level in the serum of the DNG group compared to that of the GnRHa group after 4 months of hormonal treatment. We speculated that IL-6 was suppressed by DNG treatment and thus proceeded to analyze it in more detail. Considering the time required for rearrangement of follicle cohorts, we hypothesized that the condition at the end of hormonal treatment would affect ovarian reserve; therefore, we compared serum IL-6 levels in the two groups after hormonal treatment ended (4 months). We also analyzed IL-6 and AMH levels 14 months after enrollment. However, there was no significant difference in the evaluations between the two groups (data not shown). In *an in vitro* study, Ichioka et al. suggested that DNG can influence the inflammatory response by downregulating inflammatory factors through progesterone receptors [20]. Based on the findings from previous studies and the concurrent findings from the present study, DNG is effective for ovarian reserve preservation by reducing the inflammatory response during the perioperative period and other endometriosis-related inflammation reactions [21, 22].

Another possible reason why DNG worked so effectively on ovarian reserve after ovarian cystectomy is the difference in medication effect compared to GnRHa. From our data on serum FSH levels in each group, DNG maintains higher basal secretion of FSH from the pituitary than GnRHa, and it may be effective in rearrangement of follicle cohorts derived from residual primordial follicles after ovarian cystectomy. AMH is secreted from primary to small antral follicles [23], and these follicles are FSH sensitive [24–26]. It is suggested that DNG treatment maintains basal FSH secretion, which promotes the growth of primary and small antral follicles and then increases AMH levels after medication, even before surgery. Since it takes approximately 180 days for folliculogenesis from primordial follicles to preovulatory follicles [6], the AMH levels at the time of measurement reflect the status of the medication several months before. Further analysis of the effect of DNG on ovarian follicle cohorts is needed, but the efficacy of the medicine and anti-inflammatory response may have had a positive effect on the ovarian reserve.

Our study has the limitation that although this was a randomized controlled trial, it was not double-blinded. While it might have affected the methods of the surgical approach, our study showed that there was no significant difference between the two groups in terms of hemostatis, percentage of patients who had complications with resection of follicles, suturing, and that the surgical approach was comparable between the two groups. In terms of the surgical approach, we speculated that the surgical technique did not influence the difference in AMH levels between the two groups.

## Conclusions

This study demonstrates clear evidence that perioperative DNG treatment is more beneficial than GnRHa treatment for ovarian reserve preservation in patients with endometriosis indicated for endometrioma cystectomy. Further studies are required to investigate the significance and physiology of serum AMH levels and the effect of DNG in patients with endometriomas with regard to prospective live births.

## Supplementary Information

**Additional file 1.**

## Data Availability

The datasets used and/or analyzed during the current study are available from the corresponding author on reasonable request.

## Abbreviations

GnRHa: gonadotropin-releasing hormone agonist
DNG: dienogest
AMH: anti-müllerian hormone
IL-6: interleukin-6
MRI: magnetic resonance imaging
PCOS: polycystic ovarian syndrome
rASRM: revised American Society for Reproductive Medicine
CLEIA: chemiluminescent enzyme immunoassay
SD: standard deviation
FSH: follicle stimulating hormone
IL-8: interleukin-8
TNFα: tumor necrosis factor-α
