# Supplementary material for: Impact of perioperative use of GnRH agonist or dienogest on ovarian reserve after cystectomy for endometriomas: a randomized controlled trial
Source: Reprod Biol Endocrinol. 2021 Dec 6;19:179. doi: 10.1186/s12958-021-00866-2 (PMC8647468; doi:10.1186/s12958-021-00866-2)
Supplement: Supplementary file 1 — Additional file 1. [file 12958_2021_866_MOESM1_ESM.pdf]

A

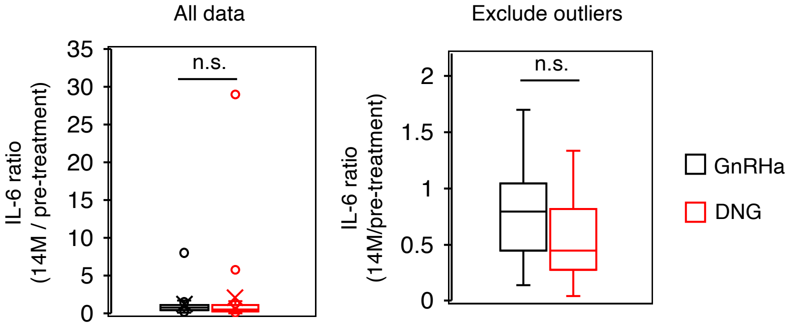

B

	0.5>	0.5<	n
GnRHa	5 (29%)	12 (71%)	17
DNG	11 (50%)	11 (50%)	22
n	16	23	*P*=0.067

C

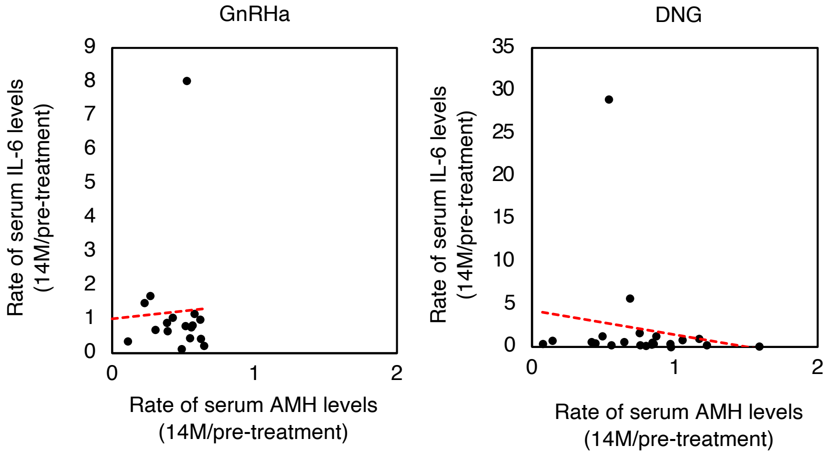

Figure legend of additional analysis

(A) The ratio of serum IL-6 levels is defined as [14M post-treatment IL-6 level / pre-treatment IL-6 level]. In the box plot, center lines show medians; box limits indicate the 25th and 75th percentiles; whiskers extend 1.5 times the interquartile range from the 25th and 75th percentiles. n.s. stands for not significant. Data were analyzed by two-tailed Student's *t*-test. (B) The number of patients whose ratio of serum IL-6 levels was under 0.5 or over 0.5 in each group. The *P*-value was calculated using the Chi-square test. (C) Scatter plot showing the correlation between AMH and IL-6 rate in each group. (GnRHa; r=0.04, P=0.09, DNG; r=0.16, P=0.30) The r is the Pearson correlation coefficient.
